# The Influence of Short Peptides on Cell Senescence and Neuronal Differentiation

**DOI:** 10.3390/cimb47090739

**Published:** 2025-09-10

**Authors:** Elena Sakhenberg, Natalia Linkova, Nina Kraskovskaya, Daria Krieger, Victoria Polyakova, Dmitrii Medvedev, Alexander Krasichkov, Mikhail Khotin, Galina Ryzhak

**Affiliations:** 1 Institute of Cytology RAS, Tikhoretski Ave., 4, 194064 St. Petersburg, Russiadaryamalikova@gmail.com (D.K.);; 2 The Department of Hospital Surgery, Belgorod National Research University, 308015 Belgorod, Russia; vopol@yandex.ru; 3 The Department of Social Rehabilitation and Occupational Therapy of the St. Petersburg Medical and Social Institute, Kondratievsky St., 72A, 195271 St. Petersburg, Russia; 4 Department of Radio Engineering Systems, Electrotechnical University “LETI”, 5F Prof. Popova St., 197022 St. Petersburg, Russia; 5 St. Petersburg Institute of Bioregulation and Gerontology, 3 Dynamo Ave., 197110 St. Petersburg, Russia

**Keywords:** induced neurons, short peptides, FetMSC, beta-galactosidase, p21, cell senescence

## Abstract

It has been previously shown that some short peptides are involved in various cellular processes, such as transcription modulation and regulation of differentiation mechanisms. In particular, the effect of peptides on the neuronal differentiation of human periodontal ligament stem cells has been demonstrated. The goal of this study was to assess the effect of KED, EDR, and AEDG short peptides in stimulating the transdifferentiation of fetal MSCs into induced neuronal cells and prevention of their senescence. We applied a novel in vitro technique for neuronal cell generation, which combines the use of microRNAs, transcription factors, and small molecules to transdifferentiate fetal mesenchymal stem cells into induced cortical neurons. It was shown that the application of AEDG and KED short peptides at the end of the transdifferentiation process decreases the expression of the cell cycle marker p21 by 15% and beta-galactosidase activity by 1.51–2.4 times. However, short peptides did not affect the expression levels of TUj-1 and LaminB1, whose expression also changes during neuronal differentiation. The experiments indicate the potential of AEDG and KED short peptides as modulators of neurogenesis and geroprotectors and suggest that they can be used as stimulators of neuronal differentiation.

## 1. Introduction

Obtaining new neuronal cells for replacement therapy in case of damage to the integrity of nervous tissue as a result of injuries, traumas, strokes, and neurodegenerative diseases remains a key challenge in contemporary neurobiology and regenerative medicine. However, cellular plasticity gradually declines during developmental progression [1]. Cellular identity is maintained and stabilized by complex genetic and epigenetic machinery to maintain the structural and functional integrity of tissues, which has long made manipulation of somatic cells difficult. It is especially difficult to obtain human neural progenitors and neurons. In the body, new neurons arise from neural stem cells during embryogenesis and persist even into adulthood in certain areas of the brain, such as the subventricular zone. The presence of neurogenesis in animals, particularly mice, has been described in detail and confirmed by numerous studies [2,3].

However, the extent and functional significance of adult neurogenesis remain under investigation, and it is still unclear whether it is sufficient to replace lost neurons and restore functional neural tissue [4,5,6]. Due to the limited regenerative potential of human nervous tissue, scientists have developed various approaches to replenish the population of nerve cells after its degeneration. With the advancement of pluripotent stem cells, including human embryonic stem cells (hESCs) and human induced pluripotent stem cells (iPSCs), new and exciting approaches for neural cell replacement are being developed [7]. While multiple studies have reported that both diseased and control iPSC-derived neurons often exhibit limited maturation beyond fetal developmental stages, as shown by transcriptomic analyses, iPSC technology remains a powerful tool for modeling early stages of human neurodevelopment and disease [8]. To overcome the constraints of iPSC-derived neuron maturation, recent progress in direct reprogramming of human somatic cells through regeneration-like pathways provides an alternative strategy for generating clinically relevant stem cells [1]. Direct reprogramming, unlike iPSC technology, allows the epigenetic signatures of the donor cell to be preserved [9,10]. There are various methods and approaches for obtaining neuronal cells and, in particular, neurons using direct reprogramming. These are based on the use of small molecules [11,12], transcription factors [13,14,15], and a combination of microRNA and transcription factors [16,17,18,19] to obtain a population of induced neurons. Neurotrophic factors such as brain-derived neurotrophic factor, nerve growth factor, glia-derived growth factor, and neurotrophins, which additionally ensure the differentiation and survival of neurons during reprogramming, are also included in the reprogramming cocktails. Various trophic factors such as GDNF have been shown to potentiate differentiation into a neuronal phenotype [20] and improve survival [21]. It has also been shown that neurotrophic factors such as BDNF and NT3 can stimulate neuronal differentiation [22].

In addition to various neurotrophic factors, polypeptide complexes and short peptides can also act as agents that stimulate neuronal differentiation and neuronal survival [23,24,25]. Short peptides, in particular Semax, increase mRNA levels for both the NGF and BDNF genes [26,27]. Previously it was found that fluorescence-marked AEDG and EDR peptides can accumulate in the cytoplasm, nucleus, and nucleolus of HeLa. This proves that AEDG and EDR peptides can penetrate into the cell and its nucleus, and, in principle, they may interact with various components of the cytoplasm and nucleus including DNA and RNA [28]. Administration of the Ala-Glu-Asp-Gly (AEDG) peptide to pluripotent cells of the Xenopus Laevis ectoderm promoted differentiation into epidermis and neural tissue. It was demonstrated that the Lys-Glu-Asp (KED) peptide and a mixture of short peptides (EDR, AEDG, KED) enhance GAP43 and nestin expression in early neuronal progenitors of human periodontal ligament stem cells (hPDLSCs). In a recent study, we also demonstrated that short peptides are able to stimulate the formation of neurites in induced neurons derived from fibroblasts of elderly donors using a direct reprogramming method [29]. Moreover, short peptides can also epigenetically regulate mechanisms of neuronal differentiation [30]. It was previously shown that short peptides can bind to the promoter region of various genes, including neuronal ones [31].

However, the effect of short peptides on the transdifferentiation process has not been previously examined. In the present study we assess whether short peptides (EDR, AEDG, KED) enhance the reprogramming procedure in fetal mesenchymal stem cells (fetMSCs). Addressing cellular stress is a crucial factor in promoting neuronal differentiation, as stress responses significantly influence cell fate and function. Short peptides have shown great potential in modulating these stress pathways, thereby facilitating neuronal survival and maturation. This study focuses on how targeting cellular stress with specific short peptides can enhance neuronal differentiation, providing insights into novel therapeutic strategies for neuroregeneration.

To evaluate the effects of these peptides, we treated cells for 10 days and analyzed key markers involved in neuronal differentiation and cellular stress. Specifically, we measured the levels of the neuronal marker beta-3-tubulin (Tuj-1), as well as lamin B1 and p21 proteins, which are known to change during differentiation. Additionally, beta-galactosidase activity was assessed. These markers were chosen because of their essential roles in monitoring cellular differentiation, neurogenesis, and stress responses, providing valuable information about the cells’ physiological state, metabolic activity, and capacity to reprogram into neurons.

The aim of this study is to evaluate the effect of EDR, KED, and AEDG peptides in stimulating transdifferentiation of FetMSCs into induced neuronal cells using a direct reprogramming technique and preventing their senescence.

## 2. Materials and Methods

### 2.1. Cell Culture

The FetMSC line was obtained from the Russian Collection of cell cultures of vertebrates (Institute of Cytology of the Russian Academy of Sciences, St. Petersburg, Russia) [32]. The FetMSC line was originally isolated from bone marrow of an early human embryo and described as multipotent mesenchymal stromal cells based on the Minimal criteria of The International Society for Cellular Therapy. Cells were cultured in DMEM/F12 media with high glucose content (Biolot, St. Petersburg, Russia) with the addition of 10% fetal bovine serum (Gibco, Waltham, MA, USA) at a temperature of 37 °C and 5% CO_2_.

### 2.2. HEK293T Cultivation and Lentivirus Production

HEK293T cells served as the line for lentivirus assembly. These cells were maintained in high-glucose DMEM medium (BioInn, St. Petersburg, Russia) supplemented with 10% fetal bovine serum (Gibco, USA) at 37 °C under a 5% CO_2_ atmosphere. For lentiviral production, HEK293T cells at 70–80% confluence were utilized. Lentiviruses were generated by co-transfecting HEK293T cells with the packaging plasmid pPAX (Addgene, #12260), the envelope plasmid pMD2.G (Addgene, #12259), and plasmids encoding the genes of interest (microRNA, transcription factors MYT1L, NeuroD2) using the cationic polymer polyethyleneimine (Polysciences, #23966). Transfections were performed in serum-free medium for 16 h, after which the medium was replaced with fresh complete medium. Virus-containing supernatants were collected 48 h post medium change. At 72 h after transfection, the conditioned medium was harvested, filtered through 0.45 μm pore size filters, and subjected to ultracentrifugation at 70,000× *g* for 2 h at 4 °C. The resulting viral pellet was resuspended in sterile phosphate-buffered saline supplemented with 20% sucrose, aliquoted, and stored at −80 °C as previously described [33].

### 2.3. Direct Reprogramming Technique

For direct reprogramming of fetMSCs into excitatory neurons, we used a modified protocol based on the method developed by A. Yoo’s group. Fibroblasts were cultured in 6-well plates until they reached full confluency. Once a monolayer was formed, the cells were transduced with four lentiviruses carrying genes encoding the reverse tetracycline-controlled transactivator, microRNA under a doxycycline-inducible promoter, and two transcription factors—MYT1L and NeuroD2. Viral transduction was performed in the presence of hexadimethrine bromide at a final concentration of 8 μg/mL. The next day, the medium was replaced with a fresh one containing 1 μM rapamycin (Selleckchem, Houston, TX, USA) for cell synchronization, as described previously [34]. After 48 h, doxycycline (DOX) was added at a final concentration of 1 μg/mL. Fibroblasts were incubated for two days, and on the third day after microRNA expression induction, DOX (1 μg/mL) and puromycin (3 μg/mL) were introduced. After another two days in the presence of antibiotics, the cells were transferred to 96-well plates pre-coated with Matrigel (Corning). Two days after reseeding, the medium was replaced with a reprogramming medium containing the same concentrations of DOX and puromycin for two rounds of selection. The reprogramming medium consisted of Neurobasal-A supplemented with 2% B-27, 0.125 mM GlutaMAX (all from Gibco, USA), 1 mM valproic acid (Sigma Aldrich, Burlington, MA, USA), 1 μM retinoic acid (Sigma Aldrich, USA), 200 μM dibutyryl cAMP (Sigma Aldrich, USA), 1 μM ISX9 (SelleckChem, Houston, TX, USA), 20 ng/mL BDNF (PeproTech, Cranbury, NJ, USA), and 20 ng/mL neurotrophin-3 (PeproTech, USA). During the first two weeks of reprogramming, 1 μM DAPT (SelleckChem, USA) was added to enhance differentiation, as described in [17]. The reprogramming process lasted 21 days. DOX was added every two days at a concentration of 1 μg/mL and every four days at 2 μg/mL. Every week, half volume of fresh reprogramming medium was added to each well. After differentiation was completed, induced neurons were treated daily with EDR, KED, or AEDR peptides with final concentration 10 μg/mL for 10 days.

### 2.4. Senescence β-Galactosidase Staining

The cells were stained using a reagent kit (Senescence Cells Histochemical Staining Kit, Sigma, USA) according to the manufacturer’s recommendations. The staining intensity was assessed according to the protocol described in an article [34]. Through use of the MathLab software (GNU Octave, v4.2.1) and accompanying plug-ins suggested by the authors of the article, the cell soma and a small area near the cell were isolated, the signal of which was taken as the background. Thus, automated calculation allows the values of the cell blue intensity (CSI) and the corresponding background (BSI) to be obtained. The logarithm (BGAL) of the quotient of these two values represents the staining intensity in relative units and allows a comparison of the activity of beta-galactosidase in the cells.

### 2.5. Immunofluorescence Staining

Before immunofluorescence staining, the cells were washed from the conditioned medium with phosphate-buffered saline (PBS) and fixed in 10% formalin for 20 min at room temperature. This was followed by a series of washes with PBS and permeabilization of cell membranes with a 0.25% Triton X-100 solution in PBS for 5 min at room temperature. Then the cells were washed with PBS 3 times and incubated in a blocking solution containing 5% bovine serum albumin (Sigma-Aldrich, St. Louis, MO, USA) diluted in PBS for 1 h at room temperature to prevent nonspecific binding of antibodies. Then the cells were incubated with primary antibodies TUJ-1 (R&D Systems, Minneapolis, MN, USA, Cat№ MAB1195, dilution: 1/2000), MAP2 (Abcam, Cambridge, UK, Cat№ ab281588, dilution: 1/1000), p21 (ELK Biotechnology, Sugar Land, TX, USA, Cat№ EA236, dilution: 1/500), and laminB1 (Novus, Chesterfield, MI, USA, Cat№ NBP2-59783, dilution: 1/100) diluted in a blocking solution overnight at +4 °C. Next, a series of washes with PBS solution was performed, after which the cells were incubated with secondary antibodies Alexa Fluor 488 goat anti-rabbit (Invitrogen, Waltham, MA, USA, Cat№ R37116, dilution: 1/1000), Alexa Fluor 555 goat anti-rabbit (Abcam, Cat№ ab150078, dilution: 1/2000), and Alexa Fluor 488 goat anti-mouse (Abcam, Cat№ ab150113, dilution: 1/2000) for 1 h at room temperature, diluted in a blocking solution. Next, a series of washes with PBS solution was performed, with DAPI staining, after which the cells were fixed on a glass slide using Mounting media (Cell Signaling, Danvers, MA, USA).

### 2.6. Western Blot

FetMSCs and induced neurons obtained from them were lysed for 30 min on ice in a RIPA buffer (ServiceBio, Wuhan, China, Cat№G2002), supplemented with a protease inhibitor at a concentration of 1 mg/mL (ThermoScientific, Waltham, MA, USA, Cat№78429). Probes were separated in a 10% SDS polyacrylamide gel, transferred onto a PVDF membrane (Bio-Rad, Hercules, CA, USA, Cat№1620264), blocked, and incubated overnight with primary antibodies to actin (ServiceBio, Cat№GB12001, dilution: 1/500), TUJ-1 (R&D Systems, Cat№ MAB1195, dilution: 1/2000), MAP2 (Abcam, cat-alog number ab ab281588, dilution: 1/2000), p21 (ELK Biotechnology, CAt№ EA236, dilution: 1/500), and laminB1 (Novus, Cat№ NBP2-59783, dilution: 1/1000). The membrane was washed several times with a PBS buffer supplemented with 0.1% polysorbate 20 (ApplyChem, Darmstadt, Germany, Cat№A4974), and then incubated with horseradish peroxidase-conjugated secondary antibodies (Bio-RAD, Cat№ 1706516, 1706515, dilution: 1/3000) for 1 h. After several washes to remove unbound secondary antibodies, the membrane was incubated in a SuperSignal West Pico PLUS Chemilumonescence Substrate (ThermoScientific, Cat№ 34577), and the signal was detected using a Chemidoc system. Analysis of the data from three batches of cultures was performed using ScionImage software (Scion Image 4.0.3.2). The mean density of each band was normalized to actin signal in the same sample and averaged. These values were normalized to fetMSC intensity for every batch of cells.

### 2.7. Statistics

Normality of distribution was assessed using the Shapiro–Wilk test. The significance level (α) was set at 0.05. Results were considered statistically significant if the probability (p) of rejecting the null hypothesis was less than α (i.e., *p* ≤ 0.05). For comparisons between two groups, the unpaired *t*-test was used in case of normal distribution and the Mann–Whitney test in case of deviation from normal distribution. For comparisons between multiple samples, one-way multivariate analysis of variance (ANOVA) was used, followed by Tukey’s post hoc test when normality was confirmed. In cases where data deviated from normal distribution, the Kruskal–Wallis test was used, followed by Dunn’s post hoc analysis. All experiments included at least three independent measurements. Results are presented as mean ± standard error of the mean (SEM) for normally distributed data and as median ± interquartile range (IQR) for non-normally distributed data.

## 3. Results

**Figure 1:**
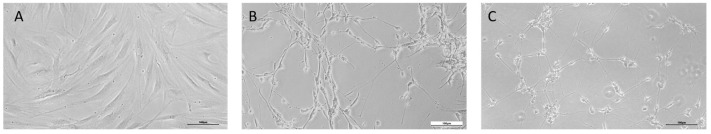
Conversion of FetMSCs into induced neurons. Representative microscopy images depicting the morphological changes after microRNA induction. (**A**)—without lentiviral transduction; (**B**)—13 days after lentiviral transduction; (**C**)—21 days after lentiviral transduction. Phase-contrast microscopy, ×10. Scale bar: 100 µm.

**Figure 2:**
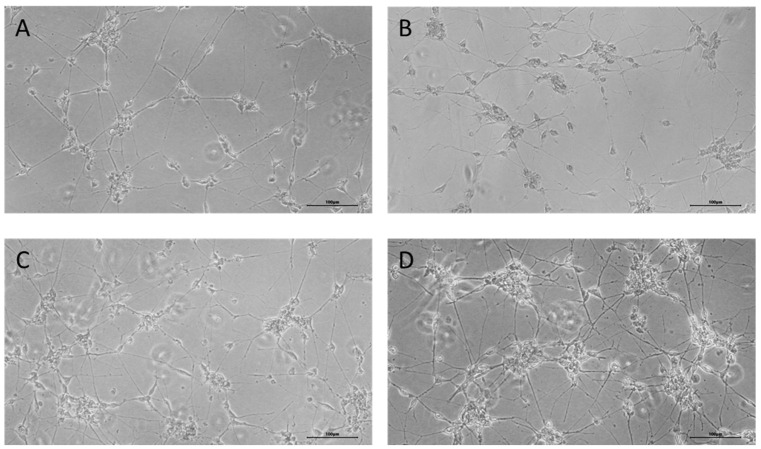
Cell morphology after application of short peptides for 10 days. Representative microscopy images of induced neurons in control cells (**A**) and after application of AEDG (**B**), EDR (**C**), and KED (**D**) peptides. Phase-contrast microscopy, ×10. Scale bar: 100 µm.

To generate induced neurons from FetMSCs, cells were transduced with lentiviruses encoding microRNA 9*-9/124 and transcription factors MYT1L and NeuroD2. After 13 days of culture in a neuronal reprogramming medium, cells displayed a morphology featuring small compact bodies and monopolar, bipolar, or, less commonly, multipolar projections (Figure 1B). After this, cells were cultured in a reprogramming medium without addition of DAPT for further maturation for 7 days. At the end of the reprogramming procedure, induced neurons displayed a complex morphology with neurite-like structures (Figure 1C). We then applied short peptides to the induced neuronal culture for 10 days to assess their effect on neuronal differentiation (Figure 2). The cells retained the complex morphological structure inherent to neuronal cells.

**Figure 3:**
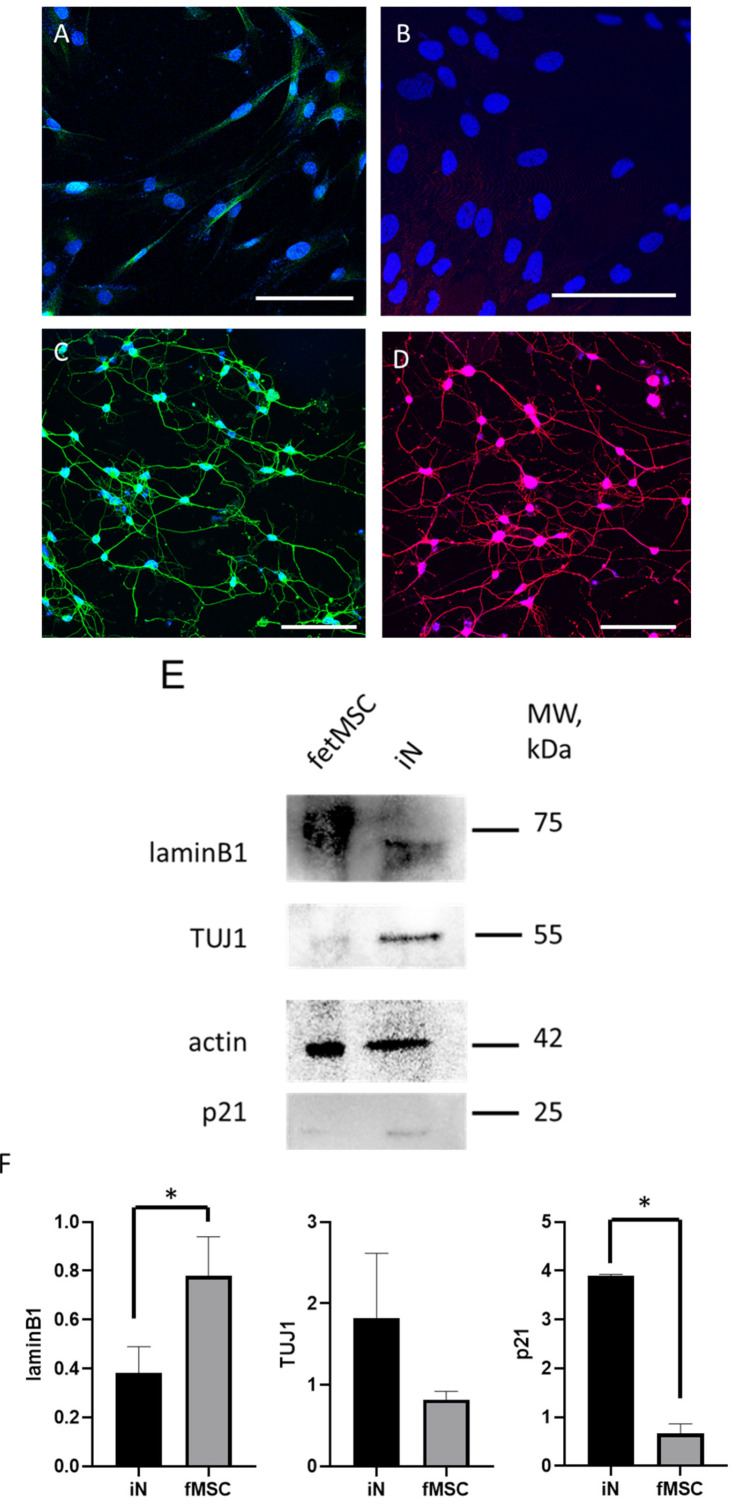
Analysis of expression of neuronal differentiation markers in induced neurons and in fetMSCs. Micrographs illustrating expression of TUj1 and MAP2 in FetMSC cell line before and after reprogramming procedure. Immunofluorescence staining with antibodies against the neuronal markers TUJ-1 (green, secondary antibody Alexa488) and MAP2 (red, secondary antibody Alexa555) in fetMSCs (**A**,**B**) and induced neurons (**C**,**D**). Nuclei visualization with DAPI staining. Confocal microscopy, ×40. Scale bar: 100 µM. (**E**) Western blot analysis of laminB1, TUJ1, and p21 expression in FetMSC cell line before and after reprogramming procedure. (**F**) Bar plots illustrating analysis of laminB1, TUJ1, and p21 expression in FetMSC cell line before and after reprogramming procedure. Data are presented as mean ± SD. * *p* < 0.05. Unpaired *t*-test.

To verify whether the cells exhibit characteristic neuronal proteins, we performed immunofluorescent staining of fetMSCs (Figure 3A,B) and the induced neurons derived from them (Figure 3D) using the following neuronal markers: microtubule-associated protein 2 (MAP2) and neuron-specific β-tubulin III (TUJ-1). Before induction, MAP2 and TUJ-1 expression is faint but becomes abundant in induced neurons. As shown in the micrographs, almost all neurons derived from fetMSCs are positively stained for neuronal markers. To evaluate the efficiency of neuronal differentiation and the associated cellular stress, we selected laminB1, p21, and TUJ1 as markers. TUJ1 is a well-established neuronal marker that indicates successful differentiation, while p21 in neurons serves as a marker of cell cycle termination and stress response. LaminB1 was chosen due to its involvement in differentiation processes, making it particularly relevant for assessing the impact of differentiation conditions on cellular integrity and stress. Expression of these markers in fetMSCs was confirmed using immunofluorescent staining (Figure 3—image from the original Western Blotting images) and using the Western blot method. Lamin B1 expression was significantly reduced in induced neurons, at 0.37 ± 0.2, compared to fetMSC expression—0.84 ± 0.3 (*p* = 0.0295). In contrast, the expression of the neuronal marker increased to 1,8± 1,5 in induced neurons compared to FetMSCs, where TUJ1 expression was 0.82 ± 0.2; however, this difference did not reach statistical significance, probably due to the elevated TUJ1 expression in fetMSCs. Also, the expression of p21 increased significantly and amounted to 3.9 ± 2.0 compared to fetMSCs, where the expression of this protein was 0.66 ± 0.3 (*p* = 0.0478).

**Figure 4:**
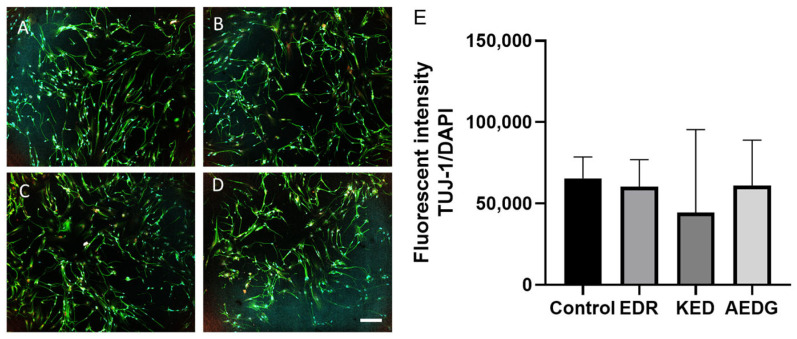
Micrographs illustrating the analysis of the level of the neuronal marker TUJ-1 in induced neurons obtained from FetMSCs in the control (**A**) and after the application of peptide bioregulators AEDG (**B**), EDR (**C**), and KED (**D**). Immunofluorescent staining with antibodies to TUJ-1 (green, secondary antibodies Alexa 488) and visualization of nuclei with DAPI dye. Spinning disk confocal microscopy, ×10. Scale bar: 100 µM. (**E**) Bar plots illustrating the expression level of TUJ-1 protein in induced neurons derived from FetMSCs. Data are presented as median ± interquartile range. Kruskal–Wallis test followed by Dunn’s post hoc analysis.

Our previous experiments showed that short peptides are able to modulate neurogenesis in neuronal progenitors derived from hPDLSC cells [35]. Hence, we first analyzed the effects of short peptides on the expression of the canonical neuronal marker TUJ-1 in induced neurons derived from FetMSCs. According to the obtained results, all three peptide bioregulators do not cause a statistically significant increase in the level of TUJ-1 protein expression in induced neurons obtained from FetMSCs compared to the control. In the control, the level of TUJ-1 expression was 65,484 ± 32,066, while after application of the AEDG peptide, the level of TUJ-1 was 60,969 ± 44,168, after application of the EDR peptide, a decrease in the protein level to 60,455 ± 62,536 was observed, and after application of the KED peptide, a decrease to 44,413 ± 31,258 was observed (Figure 4).

**Figure 5:**
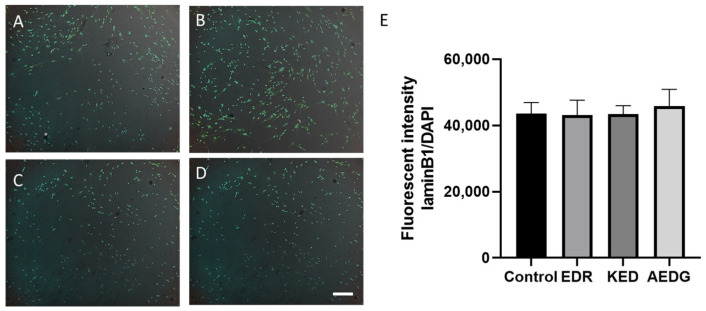
Micrographs illustrating the analysis of the laminB1 level in induced neurons obtained from FetMSCs in the control (**A**) and after the application of peptide bioregulators AEDG (**B**), EDR (**C**), and KED (**D**). Immunofluorescent staining with antibodies to laminB1 (green, secondary antibodies Alexa 488) and visualization of nuclei with DAPI dye. Spinning disk confocal microscopy, ×10. Scale bar: 100 µM. (**E**) Bar plots illustrating the expression level of laminB1 protein in induced neurons derived from FetMSCs. Data are presented as mean ± SEM.

Since the level of the neuronal marker TUJ-1 did not change by the peptide, we decided to test whether the peptides affected other proteins associated with neuronal differentiation. Nuclear lamins are another marker of cell differentiation. B-type lamins are the only ones that are observed in all cell types in the brain. Interestingly, a decrease in lamin B1 levels was observed from the differentiation phase to the maturation phase in both neurons and oligodendrocytes. It was reported that lamin B1-knockout cells exhibited severe defects in migration capacity and abnormal lamination in the cerebral cortex [36]. The balance of lamin B1 levels in the developing brain is also important. On the one hand, lamin B1 overexpression was reported to selectively reduce axonal growth, while on the other hand, lamin B1 deficiency severely impaired the length and morphology of the dendritic tree [37]. Loss of lamin B1 is a biomarker associated with aging [38], but it is not easily detected in the aged brain by immunostaining because mature neurons express low levels of lamin B1 [39]. Given the important role of lamin B1 in neuronal differentiation, we analyzed its levels in induced neurons. In induced neurons obtained from FetMSCs, the expression level of lamin B1 in the control was 43,643 ± 3304, and after application of the AEDG, EDR, and KED peptides, it did not change and was 45,838 ± 5096, 43,153 ± 4464, and 43,402 ± 2558, respectively. Thus, no statistically significant differences were found in the effect of peptide bioregulators on the level of laminB1 protein expression (Figure 5).

**Figure 6:**
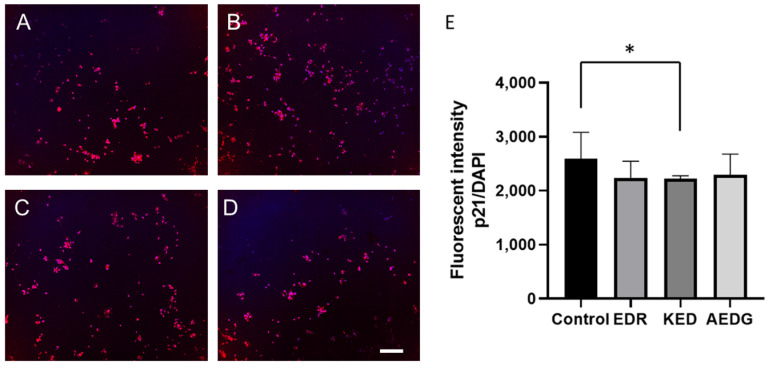
Micrographs illustrating the analysis of the p21 level in induced neurons obtained from FetMSCs in the control (**A**) and after the application of short peptides AEDG (**B**), EDR (**C**), and KED (**D**). Immunofluorescent staining with antibodies to p21 (red, secondary antibodies Alexa 555) and visualization of nuclei with DAPI dye. Spinning disk confocal microscopy, ×10. Scale bar: 100 µM. (**E**) Bar plots illustrating the expression level of p21 protein in induced neurons derived from FetMSCs before and after application of the short peptides. Data are presented as median ± interquartile range. * *p* < 0.05. Kruskal–Wallis test followed by Dunn’s post hoc analysis.

The p21 protein plays an important role in neuronal development and later in aging neurons through cell cycle regulation and DNA damage response, respectively. First, p21 significantly affects neuronal differentiation, a process by which immature cells transform into specialized neurons capable of performing unique functions in the nervous system [40]. Second, p21 is involved in cell cycle regulation during DNA damage response and DNA repair mechanisms in aging neurons [41]. Neurons can enter the cell cycle to repair DNA damage or to activate apoptosis, and p21 is associated with maintaining neurons in the G_0_ phase under these conditions, preventing inappropriate cell cycle activation and cell death [42]. P21 protein expression can increase with age in neurons. This may be related to the normal response to aging-associated DNA damage [43]. Such damage requires increased p21 levels to prevent inappropriate entry into the S phase of the cell cycle and apoptosis. In this regard, we also studied how peptides affect the level of this protein. In induced neurons obtained from FetMSC fibroblasts, the level of p21 expression in the control was 2595± 823, while after application of the KED peptide, it statistically significantly decreased to 2225± 419, and after application of the AEDG and EDR peptides, the level of the studied protein did not change and was 2234 ± 422 and 2294 ± 699, respectively (Figure 6).

**Figure 7:**
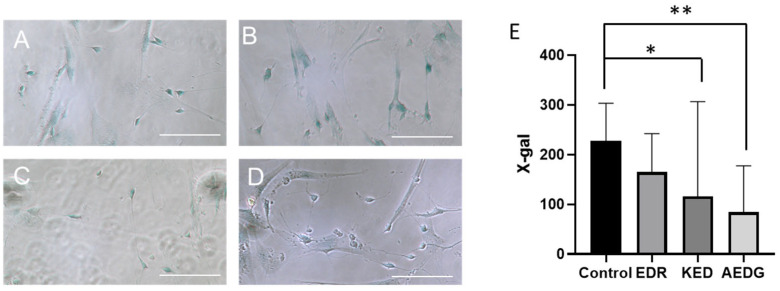
Staining of induced neurons obtained from FetMSCs for beta-galactosidase in control (**A**) and after application of AEDG (**B**), EDR (**C**), or KED (**D**) peptide. Light microscopy, ×20. Scale bar: 100 µm. (**E**) Bar plots illustrating the beta-galactosidase activity count in induced neurons derived from FetMSCs. Data are presented as median ± interquartile range. * *p* < 0.05; ** *p* < 0.01. Kruskal–Wallis test followed by Dunn’s post hoc analysis.

Another important marker characterizing aging is the activity of beta-galactosidase at pH 6.0, which is detected by histochemical staining with the addition of the X-Gal substrate [44]. Beta-galactosidase is a lysosomal enzyme that, at a certain pH, demonstrates increased activity during aging and specifically cleaves the substrate X-Gal. This marker is widely used to assess maturation and aging in most cell types including neurons both in vitro and in vivo [45,46,47]. The relative staining intensity of beta-galactosidase in the control group of induced neurons derived from FetMSC cells without peptide treatment was 227.1 ± 49 relative units. Upon application of the EDR peptide, the level did not change and was 165.1 ± 40.1 relative units. Following treatment with the KED peptide, the beta-galactosidase level significantly decreased to 116.0 ± 54.1 relative units. Similarly, after application of the AEDG peptide, the level also significantly decreased to 84.32 ± 58.9 relative units (Figure 7).

## 4. Discussion

Recently, researchers have been actively studying the influence of various bio-molecules on the processes of cell differentiation, including neurons. These processes are extremely important for neurobiology, since they can affect cell aging and their functional maturity. Moreover, small molecules and neurotrophic factors are the most promising in this regard. They induce changes in the genetic program of cells without altering the genome itself. Such an approach reduces the risk of mutations and undesired genetic modifications. In this context, short peptides, which are synthetic versions of peptides that are part of polypeptide complexes, are of particular interest. In our recent studies, short peptides AEDG, KED, and EDR were shown to participate in neuronal differentiation processes [48] and could also stimulate process formation in induced neurons obtained from fibroblasts of elderly donors using the direct reprogramming technique. In this context, peptides such as AEDG, KED, and EDR have attracted attention for their ability to modulate neuronal differentiation and protect neurons from cellular stress. Therefore, in this work, we assessed the effect of these peptides on the neuronal marker TUj1, as well as markers regulating the cell cycle and differentiation like laminB1, p21, and beta-galactosidase. Tuj-1 (β-1 tubulin): Tuj-1 is a specific marker for neurons, and its use allows the process of neurogenic differentiation to be monitored. Tuj-1 is a component of microtubules, which play an important role in the formation of the cellular skeleton and in neuritogenesis. The presence of Tuj-1 in a cell indicates that the cell has begun to differentiate into a neuron, making it an important marker for assessing the efficiency of neurogenesis and reprogramming. It was previously reported that the AEDG peptide increased the level of TUj1 mRNA during neuronal differentiation by 1.6–1.8 times in hGMSCs [49]. However, in our study, we did not observe an increase in TUJ-1 expression following short-peptide application. Therefore, we assumed that the peptides could regulate other differentiation markers, such as lam-inB1. However, even in this case, the level of this protein remained unchanged after peptide application.

It is well established that p21 plays a critical role in maintaining neurons in the G0 phase of the cell cycle, preventing their abnormal entry into the cycle and activation of apoptosis in dividing cells. Under aging or stress conditions, the level of p21 in neurons increases in response to DNA damage [43]. Interestingly, we observed that the application of the KED peptide reduced p21 levels in induced neurons. This reduction may indicate a decrease in cellular stress during the reprogramming process and a potential improvement in cell differentiation. The findings suggest that the KED peptide may help accelerate the transition of cells to a more mature state by reducing stress markers such as p21. Another important indicator of cellular aging is β-galactosidase activity, which typically increases in response to cell damage and stress. In our study, the application of AEDG and KED peptides reduced β-galactosidase activity in neurons, which points to a potential slowdown in the aging process and improved differentiation. The AEDG peptide had the most significant effect, which may highlight its unique properties in regulating cell metabolism and promoting neurogenic differentiation. This finding suggests that while these peptides influence key stress markers, they may not directly impact the reprogramming process as indicated by lamin B1 levels, probably due to high efficiency of the reprogramming protocol itself. The overall findings suggest that short peptides such as AEDG and KED may play an important role in reducing cellular stress during direct reprogramming. Their effect on reducing p21 and β-galactosidase levels may indicate increased cellular resistance to stress and an improved metabolic profile. Possible mechanisms include reduced oxidative stress, improved DNA repair, and regulation of autophagy processes. A reduction in p21 may indicate a reduced need for tight cell cycle control, due to either increased neuronal differentiation or a more favorable cellular environment. Similar effects have been previously observed for antioxidants (e.g., resveratrol) [50] and autophagy modulators (e.g., rapamycin), supporting the hypothesis of a protective effect of peptides. Thus, it can be assumed that the peptides not only promote the maturation of induced neurons, but also protect them from stress factors by reducing the level of p21 and β-galactosidase and maintaining a “younger” metabolic profile of cells. Further research is needed to explore the mechanisms through which these peptides modulate cellular stress.

## Data Availability

Dataset available on request from the authors.
